# Occult lymph node metastases in patients without residual muscle-invasive bladder cancer at radical cystectomy with or without neoadjuvant chemotherapy: a nationwide study of 5417 patients

**DOI:** 10.1007/s00345-021-03839-7

**Published:** 2021-09-28

**Authors:** L. M. C. van Hoogstraten, E. J. van Gennep, L. A. L. M. Kiemeney, J. A. Witjes, C. S. Voskuilen, M. Deelen, L. S. Mertens, R. P. Meijer, J. L. Boormans, D. G. J. Robbrecht, L. V. Beerepoot, R. H. A. Verhoeven, T. M. Ripping, BlaZIB Study Group, B. W. G. van Rhijn, K. K. H. Aben, T. J. N. Hermans

**Affiliations:** 1 Department of Research and Development, Netherlands Comprehensive Cancer Organisation, Utrecht, The Netherlands; 2 Department of Urology, Leiden University Medical Center, Leiden, The Netherlands; 3 Department of Urology, Radboud University Medical Center, Nijmegen, The Netherlands; 4 Radboud University Medical Center, Radboud Institute for Health Sciences, Nijmegen, The Netherlands; 5 Department of Urology, Netherlands Cancer Institute—Antoni Van Leeuwenhoek Hospital, Amsterdam, The Netherlands; 6 Department of Urology, Maastricht University Medical Center+, Maastricht, The Netherlands; 7 Department of Oncological Urology, University Medical Center Utrecht, Utrecht, The Netherlands; 8 Department of Urology, Erasmus University Medical Center Rotterdam, Rotterdam, The Netherlands; 9 Department of Medical Oncology, Erasmus Medical Center Cancer Institute, Rotterdam, The Netherlands; 10 Department of Medical Oncology, Elisabeth-TweeSteden Hospital, Tilburg, The Netherlands; 11 Department of Medical Oncology, Cancer Center Amsterdam, Amsterdam University Medical Centers, University of Amsterdam, Meibergdreef 9, 1105 AZ Amsterdam, The Netherlands; 12 Department of Surgical Oncology (Urology), Netherlands Cancer Institute-Antoni Van Leeuwenhoek, Amsterdam, The Netherlands; 13 Department of Urology, Catharina Hospital Eindhoven, Eindhoven, The Netherlands

**Keywords:** Bladder cancer, Neoadjuvant chemotherapy, Downstaging, Lymph node metastases, Radical cystectomy

## Abstract

**Purpose:**

Little is known about the prevalence of occult lymph node metastases (LNM) in muscle-invasive bladder cancer (MIBC) patients with pathological downstaging of the primary tumor. We aimed to estimate the prevalence of occult LNM in patients without residual MIBC at radical cystectomy (RC) with or without neoadjuvant chemotherapy (NAC) or neoadjuvant radiotherapy (NAR), and to assess overall survival (OS).

**Methods:**

Patients with cT2-T4aN0M0 urothelial MIBC who underwent RC plus pelvic lymph node dissection (PLND) with curative intent between January 1995–December 2013 (retrospective Netherlands Cancer Registry (NCR) cohort) and November 2017–October 2019 (prospective NCR-BlaZIB cohort (acronym in Dutch: BlaaskankerZorg In Beeld; in English: Insight into bladder cancer care)) were identified from the nationwide NCR. The prevalence of occult LNM was calculated and OS of patients with <(*y*)pT2N0 vs. <(*y*)pT2N+ disease was estimated by the Kaplan–Meier method.

**Results:**

In total, 4657 patients from the NCR cohort and 760 patients from the NCR-BlaZIB cohort were included. Of 1374 patients downstaged to  <(*y*)pT2, 4.3% (*N* = 59) had occult LNM 4.1% (*N* = 49) of patients with cT2-disease and 5.6% (*N* = 10) with cT3-4a-disease. This was 4.0% (*N* = 44) in patients without NAC or NAR, 4.5% (*N* = 10) in patients with NAC, and 13.5% (*N* = 5) in patients with NAR but number of patients treated with NAR and downstaged disease was small. The prevalence of  <(*y*)pT2N+ disease was 4.2% (*N* = 48) in the NCR cohort and 4.6% (*N* = 11) in the NCR-BlaZIB cohort. For patients with  <(*y*)pT2N+ and  <(*y*)pT2N0, median OS was 3.5 years (95% CI 2.5–8.9) versus 12.9 years (95% CI 11.7–14.0), respectively.

**Conclusion:**

Occult LNM were found in 4.3% of patients with cT2-4aN0M0 MIBC with (near-) complete downstaging of the primary tumor following RC plus PLND. This was regardless of NAC or clinical T-stage. Patients with occult LNM showed considerable worse survival. These results can help in counseling patients for bladder-sparing treatments.

**Supplementary Information:**

The online version contains supplementary material available at 10.1007/s00345-021-03839-7.

## Introduction

The standard treatment for clinically node-negative muscle-invasive bladder cancer (MIBC) is radical cystectomy (RC) and pelvic lymph node dissection (PLND) with cisplatin-based neoadjuvant chemotherapy (NAC) in fit patients [1]. An alternative for RC is trimodality therapy (TMT) [1]. Transurethral resection (TUR) with or without external beam radiation therapy (EBRT) is considered inferior to RC or TMT [1, 2], whereas TUR with or without systemic chemotherapy has the potential to be curative in selected cases [3–5]. The prevalence of occult lymph node metastases (LNM) at RC plus PLND is approximately 25% and as such, PLND is associated with improved survival in these patients [6, 7]. In contrast, PLND or treatment of the lymph nodes is not part of the TMT protocol [2].

A recent Dutch population-based study including 4508 patients with cT2N0M0 urothelial MIBC showed that downstaging to non-MIBC was present in 25% after upfront RC and in 43 and 33% after NAC and neoadjuvant radiation (NAR), respectively [8]. In general, it is still not possible to accurately predict downstaging by TUR. Therefore, RC with PLND remains the standard of care. In selected cases or due to patient refusal, one might not always proceed to RC, CMR or EBRT [3, 4]. A clinical complete response after TUR-only or TUR combined with systemic chemotherapy cannot reliably be concluded based on a combination of Re-TUR, negative cytology and cross-sectional imaging. However, these diagnostics are often performed in daily practice in attempting to confirm a so called “pT0-status” in patients who prefer bladder preservation [3, 4, 9, 10]. In these patients, PLND for the assessment of nodal invasion is not routinely performed and the prevalence of occult metastatic disease and the potential role of PLND in this particular group has not been clearly demonstrated [11].

In a recent retrospective cohort of patients treated with NAC plus RC, 4.9 and 5.4% of patients with ypT0 and ypTa/is/1 disease had occult LNM [11]. This was irrespective of NAC or initial clinical T-stage. To our knowledge, other studies on this subject are not available. Therefore, the aim of this population-based study is to estimate the prevalence of occult LNM in patients without residual MIBC at RC, stratified by treatment with or without NAC and to assess OS in patients with and without occult LNM.

## Materials and methods

### Patients

Patients diagnosed with cT2-4aN0M0 urothelial bladder carcinoma (BC) who underwent RC plus PLND with or without NAC or neoadjuvant radiotherapy (NAR), between January 1st 1995 and December 31st 2013 (retrospective NCR cohort, data already available from Hermans et al. [8]) and between November 1st 2017 and October 31st 2019 (prospective NCR-BlaZIB cohort) were selected from the Netherlands Cancer Registry (NCR). The NCR-BlaZIB cohort consisted of patients included in the ongoing Dutch nationwide population-based prospective BlaZIB study (BlaaskankerZorg In Beeld, translation: Insight into Bladder Cancer Care) [12], which is embedded in the NCR. Patients who underwent a partial cystectomy or salvage cystectomy, or in whom PLND was not performed were excluded. Patients with histology other than UC as the main component were also excluded (Supplementary Fig. 1).

### The Netherlands Cancer Registry

The NCR is a nationwide population-based registry collecting data on all newly diagnosed malignancies in the Netherlands. Identification is mainly based on notification from the nationwide network and registry of histopathology and cytopathology in the Netherlands (PALGA) [13]. Well-trained data managers of the NCR collect clinical data on predefined patient, tumor, and treatment characteristics from the individual patient files at each hospital. In the NCR, topography and morphology are classified according to the International Classification of Diseases for Oncology (ICD-O) [14]. Tumor stage is classified according to the TNM system [15]. Clinical staging was based on physical examination, findings at cystoscopy and TUR, computed tomography (CT-) scan of the abdomen/pelvis and chest imaging (at least a chest X-ray).

In a previous study, all pathology reports of patients from the NCR cohort 1995–2013 were reviewed (TH, MD, CV, LM) after linkage with PALGA since pathological downstaging at RC to non-MIBC was not registered in the NCR as a standard item before 2017 [8]. For the NCR-BlaZIB cohort, information on pathological downstaging was prospectively collected. Changes in TNM classifications over time (e.g., changes within pT2-stage) were irrelevant for our study outcomes [15]. Due to changes in the classification for nodal disease, it was only possible to categorize patients into node-negative (pN0) and node-positive disease (pN+).

### Statistical analyses

The numbers and percentages of occult LNM in patients without and with (*y*) NAC and complete [(y)pT0] or partial downstaged [(*y*)pTa/is/1] primary tumors were calculated. The Kaplan–Meier method was applied to calculate median overall survival (OS) in patients with (*y*)pT0N0 vs. (*y*)pT0N+ disease and  <(*y*)pT2N0 vs. <(*y*)pT2N+ disease. Due to the limited number of patients, it was not possible to further stratify results by NAC or NAR. Date of RC was taken as start of follow-up. End of follow-up was defined as last date of follow-up or death, whatever came first. Log-rank tests were used to compare survival distributions. Statistical analyses were performed with SAS version 9.4 (SAS Institute, Cary, North Carolina, USA). *P-*values  < 0.05 were considered statistically significant.

## Results

In total 5417 patients with cT2-4aN0M0 urothelial MIBC who underwent RC and PLND were analyzed. From the retrospective NCR cohort, 4657 patients were included and from the prospective NCR-BlaZIB cohort, 760 patients were included (Supplementary Fig. 1). Compared to the NCR cohort, patients in the NCR-BlaZIB cohort were older (70 versus 67 years) and more often had locally advanced disease (cT3/4a in 29.7% versus 18.2%) (Supplementary Table 1). In the earlier NCR cohort, NAC and NAR were applied in 6.4% (*N* = 298) and 2.2% (*N* = 104) of patients, respectively. This was 28.3% (*N* = 215) and 0.5% (*N* = 4) in the NCR-BlaZIB cohort.

In 18.7% (*N* = 1013) of all PLND specimens LNM were found. In 1,374 patients downstaged to  <(*y*)pT2, 4.3% (*N* = 59) had occult LNM. In patients downstaged to (*y*)pT0 or (*y*)pTa/is/1, LNM were present in 4.1% (*N* = 33) and 4.6% (*N* = 26), respectively (Table 1). In patients with cT2 and cT3-4a disease downstaged to  <(*y*)pT2, LNM were present in 4.1% (*N* = 49) and 5.6% (*N* = 10) (*p* = 0.3705), respectively. Stratification by NAC (upfront RC vs. NAC + RC) resulted in comparable percentages of  <ypT2N+ and  <pT2N+ disease in 4.5% (*N* = 10) and 4.0% (*N* = 44) of patients (*p* = 0.7093). In 108 patients who received NAR, 5 out of 37 (13.5%) had LNM with  <ypT2 at RC. The prevalence of  <(*y*)pT2N+ disease was similar over time, 4.2% (*N* = 48) in the NCR cohort and 4.6% (*N* = 11) in the NCR-BlaZIB cohort.

**Table 1:** The prevalence of occult lymph node metastases in patients with cT2-4aN0M0 urothelial bladder cancer without evidence of residual muscle-invasive disease at radical cystectomy

	pN0	pN1-3	Total
All patients			
pT0	781	33 (4.1%)	814
pTa/is/1	534	26 (4.6%)	560
< pT2	1315	59 (4.3%)	1374
cT2 (*N* = 4342)			
pT0	673	25 (3.6%)	698
pTa/is/1	472	24 (4.8%)	496
< pT2	1145	49 (4.1%)	1194
cT3-4a (*N* = 1075)			
pT0	108	8 (6.9%)	116
pTa/is/1	62	2 (3.1%)	64
< pT2	170	10 (5.6%)	180
No NAC or NAR (*N* = 4798)			
pT0	585	20 (3.3%)	605
pTa/is/1	486	24 (4.7%)	510
< pT2	1071	44 (4.0%)	1115
NAC* (*N* = 513)			
ypT0	171	8 (4.5%)	179
ypTa/is/1	42	2 (4.6%)	44
< ypT2	213	10 (4.5%)	223
NAR* (*N* = 108)			
ypT0	25	5 (16.7%)	30
ypTa/is/1	7	0 (0%)	7
< ypT2	32	5 (13.5%)	37

*NAC* neoadjuvant chemotherapy, *NAR* neoadjuvant radiotherapy

^*^Two patients received both NAC and NAR

**Fig. 1:**
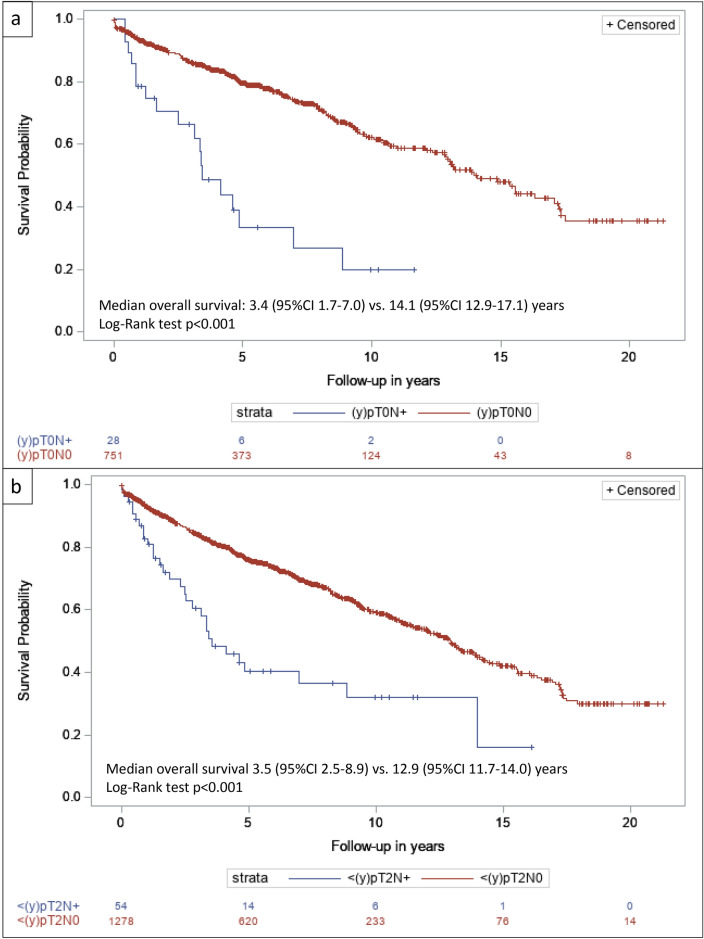
**a**–**b** Overall survival of patients with and without occult lymph node metastases in cT2-4aN0M0 urothelial bladder cancer without evidence of residual bladder cancer [(*y*)pT0] at radical cystectomy (**a**) or residual muscle-invasive disease [(*y*)pT0/a/is/1] at radical cystectomy (**b**)

Patients with LNM following complete downstaging of the primary tumor [(*y*)pT0N+] showed inferior OS versus patients with complete downstaging without LNM [(*y*)pT0N0] (*p* < 0.001). Median OS was 3.4 (95% CI 1.7–7.0) vs. 14.1 years (95% CI 12.9–17.1) (Fig. 1a). This association was also seen in patients with downstaging to non-MIBC [<(*y*)pT2] (*p* < 0.001). The median OS was 3.5 (95% CI 2.5–8.9) vs. 12.9 (95% CI 11.7–14.0) years (Fig. 1b). Groups were too small to stratify by use of neoadjuvant treatment (only 10 patients with  <pT2N+ after NAC). For the NCR cohort, median follow-up was 3.6 years with follow-up censored at 1 February 2017. For the NCR-BlaZIB cohort, median follow-up was 0.9 years with follow-up censored at 1 February 2020.

## Discussion

Pathological downstaging to non-MIBC or pT0 at RC is a favorable prognostic factor. Nevertheless, we showed that LNM are present in 4.1 and 4.6% of patients with a complete downstaging [(*y*)pT0] or near-complete downstaging [(*y*)pTa/is/1] of the primary tumor. This was regardless of the use of NAC. Moreover, these LNM were significantly associated with worse OS.

A systematic review by Bruins et al. indicated that any kind of PLND at RC is associated with beneficial OS versus no PLND [7]. Despite the low level of evidence, current guidelines recommend PLND as standard practice in combination with RC [1]. In patients who are not fit enough for RC, refuse RC or prefer a bladder-sparing approach, treatment of the pelvic lymph nodes is usually not performed. In the 2019 EAU-ESMO Consensus Statements on the management of advanced and variant BC, 64% of the experts agreed that in cN0-disease, PLND in case of bladder preservation is not recommended [16]. In contrast, a similar percentage of experts agreed on radiation of the pelvic lymph nodes in case of trimodality treatment [16]. However, given the limited evidence available in current literature no definitive consensus could be reached for both statements.

In the randomized BC2001 trial [2], disease-free survival (DFS) was compared between patients with cT2-4aN0M0 BC who underwent chemoradiotherapy (CMR) versus ERBT alone (radiation was confined to the bladder in both groups). The rate of lymph node relapses was not as high as might have been expected from surgical staging in RC cohorts, e.g., 4.9% (*n* = 9) in the CMR group and 6.7% (*n* = 12) in the ERBT-only group [2]. In another randomized chemoradiotherapy trial (cT2-4N0M0), in which radiation of the whole pelvis was compared to radiation of the bladder alone, pelvic lymph node recurrences occurred in 15.8% (15/95) and 17.6% (16/91) of patients, respectively [17]. With a median follow-up of 5 years, OS and DFS did not significantly differ between groups. In the bladder only group the first draining lymph nodes might also have been irradiated since in general a 2 cm margin around the bladder is taken. Of note, differences in pelvic lymph node recurrences between the above mentioned trials might be due to the higher percentage of T2 patients at baseline in the BC2001 trail (83 vs. 46%) [2, 17]. Several other, mostly retrospective studies on bladder-preserving strategies without EBRT or TMT following TUR (e.g., regimens of TUR—NAC—Re-TUR) did not report on the prevalence of LNM during follow-up and thereby do not address the potential role of PLND or treatment of the pelvic lymph nodes in such cases [18]. Since data on the survival effect of PLND in bladder-sparing approaches are not available, it would be interesting to compare morbidity and oncological outcomes for no treatment versus radiation versus minimal-invasive surgery for the pelvic lymph nodes in patients with MIBC undergoing bladder-preserving therapies with and without chemotherapy.

In the context of our study, it is important to note that the prevalence of LNM cannot simply be translated to the clinical scenario of selected patients with a presumed ‘pT0 status’ after a TUR with or without NAC and Re-TUR. Given significant discrepancies in residual tumor and LNM rates between a presumed ‘(*y*)pT0 status’ and a confirmed pT0-disease in RC specimens [9, 10], our results might indicate an underestimation of the prevalence of LNM in patients who are treated with TUR and/or NAC only. For example, in our RC cohort, occult LNM were present in 13% of patients with pT2-disease. Moreover, in our study PLND templates were not available, which might further underestimate the true prevalence of LNM. An earlier published NCR study indicated evidence of PLND template extension in more recent study years, as was shown by a higher number of LNM in patients with comparable clinical disease characteristics over time [19]. In line with these findings, pelvic and sentinel lymph node mapping studies in BC confirm that a limited versus an extended PLND does not capture all draining lymph nodes and thus might lead to a false negative ‘pN0 status’[20, 21]. It is, therefore, likely that the true prevalence of LNM in patients with a presumed ‘pT0 status’ before RC is higher than the 5% which was found in both the study of Nassiri et al. [11] and our study. This assumption might favor the harm to benefit ratio to perform a diagnostic PLND. Although the survival benefit of PLND in this particular group of patients is unknown, the outcome may guide adjuvant treatment. The CheckMate 274 study showed improved DFS in patients with lymph node-positive disease after NAC plus RC and PLND treated with adjuvant nivolumab [22].

It can be questioned if there are viable alternatives to a PLND or tools to select patients for whom a PLND is appropriate. The vast majority of patients in our database was staged with a contrast enhanced CT of the abdomen and a CT or conventional X-ray of the chest. Mertens et al. recently showed that by use of a FDG-PET-CT, 21% of patients were upstaged to non-localized disease [23]. Half of this group was upstaged due to regional nodal metastases. The other half had supraregional nodal or distant metastases. Clinical management changed in 13.5% of patients as a result of upstaging defined by FDG-PET-CT [23]. More sensitive imaging modalities, like FDG-PET-CT, might better select patients for PLND treated within a bladder-sparing treatment protocol. Still, according to a systematic review and meta-analysis by Ha et al. the pooled sensitivity for the detection of LNM by FDG-PET-CT was only 57% [24]. One could also argue if a sentinel node (SN) procedure could have a role in whether or not to proceed with PLND, thereby minimizing surgical risks. In BC, the reported SN detection rates range from 81 to 92%. However, in initial validation studies false negative rates up to 19% were reported [25]. In a recent single center study, Zarifmahmoudi et al. reported a SN detection rate of 85% and a false negative rate as high as 42% [26]. However, another MIBC study concluded that SN detection played no role in staging of nodal disease since the vast majority of LNM were detected in the non-sentinel lymph nodes [27]. The high number of false negatives would, therefore, lead to understaging if one does not proceed with PLND if the outcome of the SN is negative. Altogether, prospective research in promising imaging modalities and minimally invasive diagnostics is needed to further clarify the role of PLND in bladder-sparing treatment protocols in which PLND is not standard of care.

The presence of circulating tumor cells (CTCs) in patients with muscle-invasive bladder cancer is another promising area of research. A recently presented abstract from the CirGuidance study, evaluating the role of CTCs in relation to response to NAC, showed promising results: CTC-positive patients had better overall survival when they received NAC [29]. However, the full content of this study is not yet published. It would be of interest to know whether the presence of CTCs is also predictive for occult LNM in patient with and without NAC.

Our study is subject to several limitations. In the earlier cohort, data were retrospectively collected in contrast with the more recent prospective NCR-BlaZIB cohort. Despite the high number of RCs, the group of patients with (near) complete downstaging and the presence of LNM remained low. Also, information on neoadjuvant treatment was limited. In case of NAC, exact regimens and the number of cycles were unknown. This was the same for radiation schemes in the NAR-group. Recent changes in preoperative ﻿diagnostic modalities, e.g., the use of more sensitive imaging like FDG-PET scans might result in a Will Rogers phenomenon [28]. Unfortunately, our databases had no information available regarding the use of FDG-PET scans versus conventional CT scans. Therefore, we could not assess the primary study outcome stratified by different preoperative imaging modalities. However, since the prevalence of occult LNM was similar between cohorts (NCR cohort: 4.2%, NCR-BlaZIB cohort: 4.6%) we expect the impact of such stage migration to be minimal. No information was available on the extent of the PLND templates. Since a limited PLND was often performed in the past, it is likely that we underestimated the true prevalence of occult nodal metastasis in this study. This may, however, further strengthen the potential role of PLND in selected patients who do not undergo RC. In addition, this emphasizes the need for future research to evaluate, for example, the extent of the PLND template, lymph node density in positive cases and extracapsular extension in lymph nodes and their effects on prognosis and adjuvant treatments. Also, it will be important to identify risk factors predicting the presence of occult LNM after downstaging of the primary tumor (e.g., lymphovascular invasion, perineural spread, Ki-67 index on TURBT), as this might influence treatment decision-making as well. Despite these limitations, this is the second large nationwide database study to report on the prevalence of LNM in the patients with bladder cancer that were downstaged to (y)pT0 or (y)pTa/is/1 disease in the RC specimen.

## Conclusion

After RC and PLND for cT2-4aN0M0 urothelial BC, occult LNM occur in 4.3% of patients with a (near)-complete downstaging of the primary tumor. This was regardless of NAC or initial clinical T-stage. Patients with occult LNM showed considerable worse survival. The risk of occult LNM should be considered and discussed with patients opting for bladder-sparing treatment. Future research, therefore, should address the diagnostic and therapeutic value of PLND in patients with MIBC undergoing bladder-sparing treatment protocols (e.g., TUR-only ± NAC, EBRT or TMT). Consequently, the outcome of PLND may have implications for radiation field extension, adjuvant treatment with chemotherapy or immune checkpoint inhibitors.

## Supplementary Information

Below is the link to the electronic supplementary material.

Supplementary file1 (PDF 123 kb)

## Data Availability

All data used for this study can be requested from the NCR. All data requests are reviewed by the supervisory committee of the NCR for compliance with the NCR objectives and (inter)national (privacy) regulation and legislation.

## Code availability

Code used for this study can be made available post publication by the authors upon request.
