# Supplementary material for: Occult lymph node metastases in patients without residual muscle-invasive bladder cancer at radical cystectomy with or without neoadjuvant chemotherapy: a nationwide study of 5417 patients
Source: World J Urol. 2021 Sep 28;40(1):111–8. doi: 10.1007/s00345-021-03839-7 (PMC8813846; doi:10.1007/s00345-021-03839-7)
Supplement: Supplementary file 1 — Supplementary file1 (PDF 123 kb) [file 345_2021_3839_MOESM1_ESM.pdf]

Supplementary material

Supplementary Figure 1. Inclusion of patients with cT2-T4aN0M0 urothelial bladder carcinoma who underwent radical cystectomy followed by pelvic lymph node dissection in the Netherlands, stratified per cohort (NCR: 1995-2013, and NCR-BlaZIB: November 2017-October 2019)

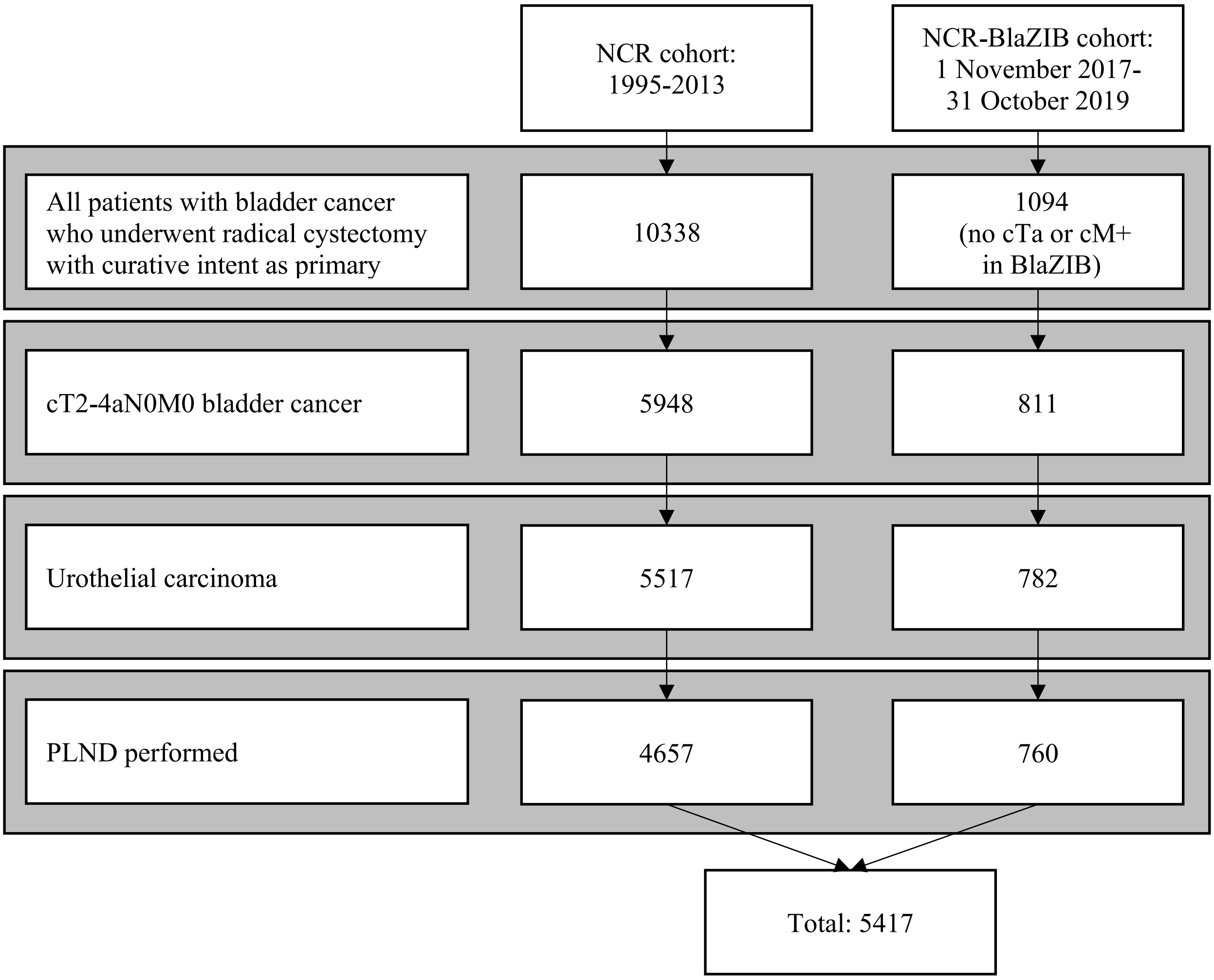

Supplementary Table 1. Baseline characteristics of patients with cT2-4aN0M0 urothelial bladder cancer without evidence of residual muscle-invasive disease at radical cystectomy

				Cohort			
		Total		NCR (1995-2013)		NCR-BlaZIB (2017-2019)	
		N	(%)	N	(%)	N	(%)
Total		5417	(100.0)	4657	(86.0)	760	(14.0)
Gender							
	Male	4116	(76.0)	3565	(76.6)	551	(72.5)
	Female	1301	(24.0)	1092	(23.4)	209	(27.5)
Age at diagnosis (median, IQR)		67.0	(60.0-73.0)	67.0	(60.0-73.0)	70.0	(63.0-75.0)
Age at diagnosis							
	<60 years	1244	(23.0)	1125	(24.2)	119	(15.7)
	60-70 years	2003	(37.0)	1757	(37.7)	246	(32.4)
	70-80 years	1882	(34.7)	1533	(32.9)	349	(45.9)
	80+ years	288	(5.3)	242	(5.2)	46	(6.1)
Clinical T-stage							
	cT2	4342	(80.2)	3808	(81.8)	534	(70.3)
	cT3-4a	1075	(19.8)	849	(18.2)	226	(29.7)
Neo-adjuvant chemotherapy							
	Yes	513	(9.5)	298	(6.4)	215	(28.3)
	No	4904	(90.5)	4359	(93.6)	545	(71.7)
Neo-adjuvant radiotherapy							
	Yes	108	(2.0)	104	(2.2)	4	(0.5)
	No	5309	(98.0)	4553	(97.8)	756	(99.5)

Abbreviations: IQR, Interquartile range.
